# Supplementary figures and images for: Wastewater-based epidemiology surveillance as an early warning system for SARS-CoV-2 in Indonesia
Source: PLoS One. 2024 Jul 18;19(7):e0307364. doi: 10.1371/journal.pone.0307364 (PMC11257287; doi:10.1371/journal.pone.0307364)

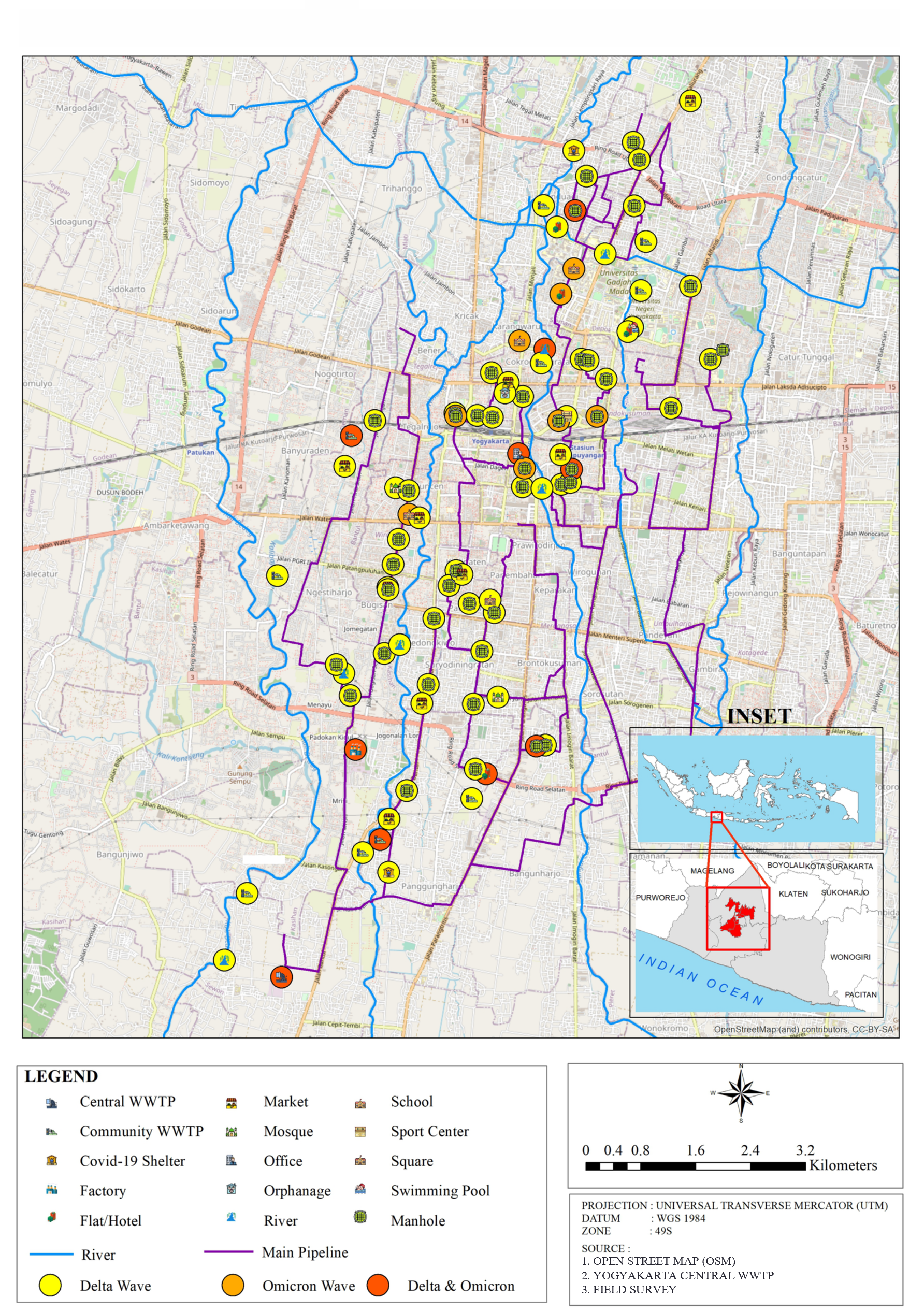

Supplement: S1 Fig — During the Delta wave, locations included manholes (n = 40), permanent residences (n = 3), temporary residences (n = 3), public facilities (n = 20), offices (n = 3), rivers (n = 6), WWTPs (n = 12). During the Omicron wave, locations included manholes (n = 7), permanent residences (n = 2), offices (n = 2), schools (n = 3), rivers (n = 1), WWTPs (n = 3). All manholes were connected to the central WWTPs. (TIF) [file pone.0307364.s001.tif]

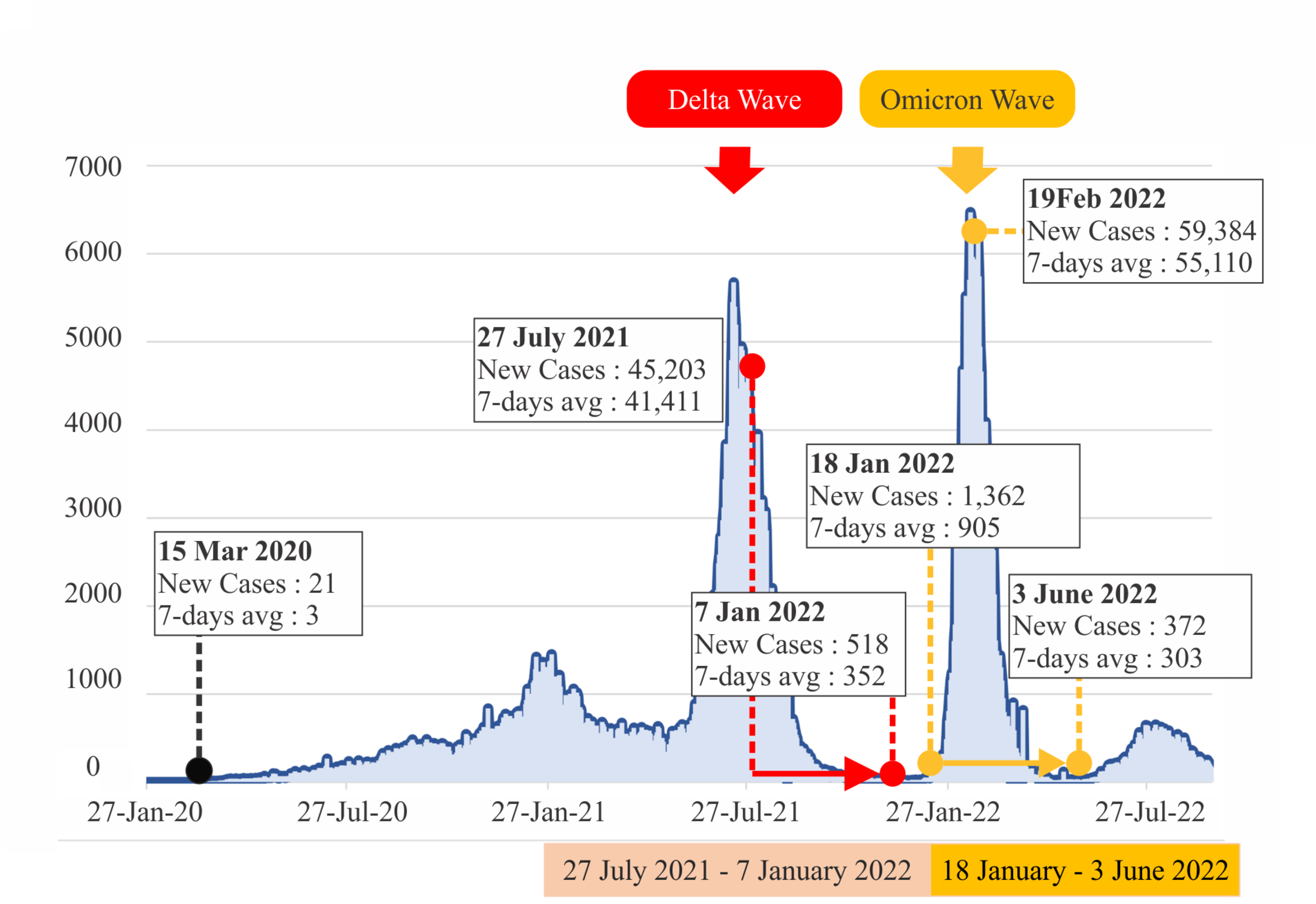

Supplement: S2 Fig — Source: https://ourworldindata.org/covid-vaccinations?country=IDN, accessed 27 July 2022. (TIF) [file pone.0307364.s002.tif]

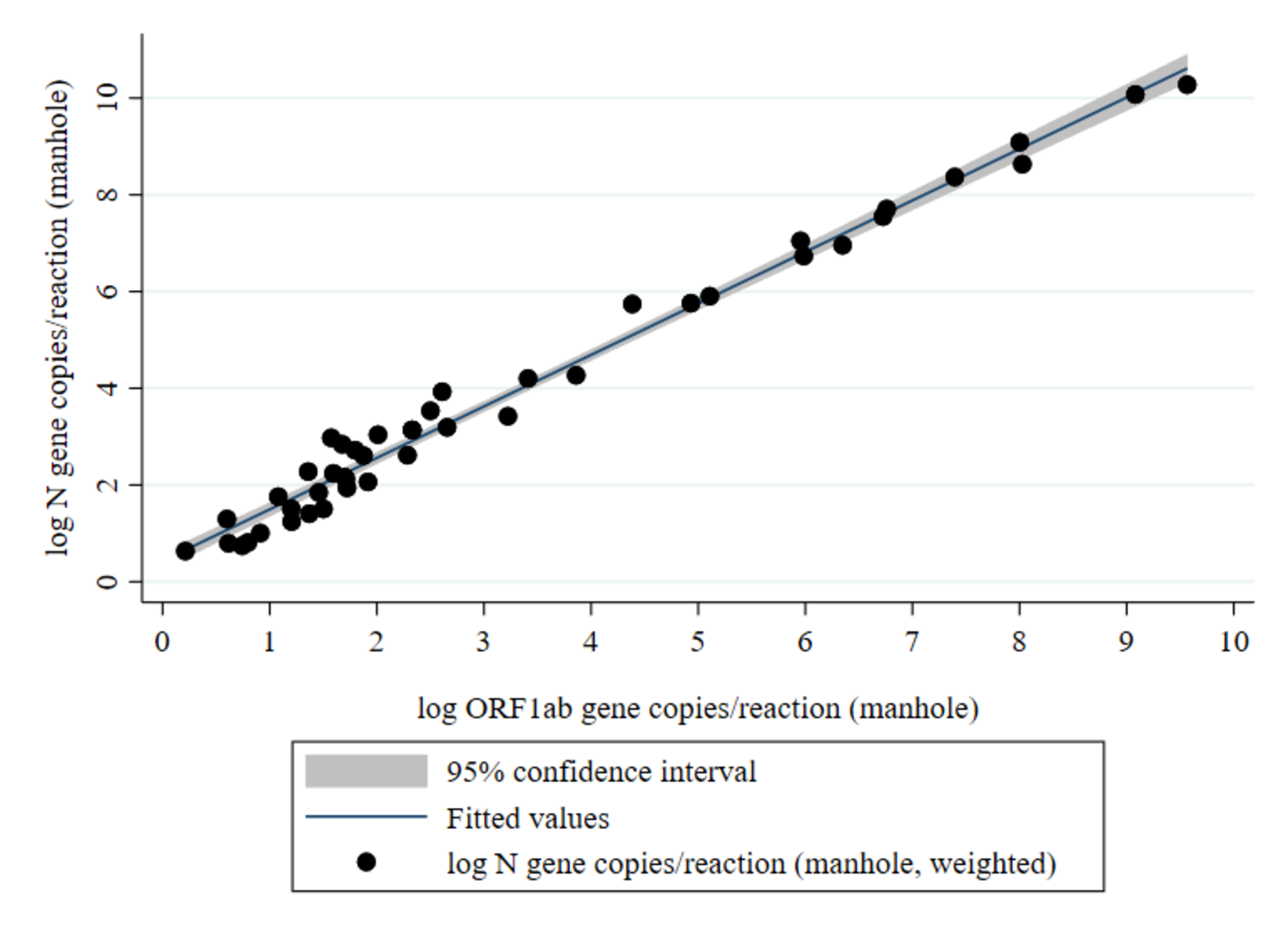

Supplement: S3 Fig — Recovery adjusted, r = 0.972, P<0.001. (TIF) [file pone.0307364.s003.tif]
