# Supplementary material for: Wastewater-based epidemiology surveillance as an early warning system for SARS-CoV-2 in Indonesia
Source: PLoS One. 2024 Jul 18;19(7):e0307364. doi: 10.1371/journal.pone.0307364 (PMC11257287; doi:10.1371/journal.pone.0307364)
Supplement: S1 File — (DOCX) [file pone.0307364.s004.docx]

# S1 File. Extraction and RT-qPCR protocol.

1. Sample Collection

Wastewater samples were collected using either a grab or passive sampling method. For the grab method, a sterile bottle pre-labelled with a barcode was immersed into the water to a depth of around 20 – 30 cm until the bottle was filled to the bottom of the neck, allowing about 1 cm of air. A 500 mL wastewater sample was collected from manholes, and a 2,000 mL sample was collected from rivers or the swimming pool.

A torpedo-style passive sampler with multiple entry points (front, top, sides, and bottom) was used to collect the wastewater sample over a more prolonged period. This passive sampler was obtained from Monash University (DMcC) and has been validated elsewhere.^15-17^ The passive sampler was deployed at the site and retrieved 24 hours later. The passive sampling material used inside these torpedoes were 47 mm diameter, 0.45µm pore size, cellulose nitrate electronegative membrane (Sartorius, Germany).

Twenty grams of the soil samples from selected NST sites including traditional markets, offices, and city squares were collected and inserted into a labeled zip lock bag. Within four hours of collection, samples were placed in the cool box and transferred on ice at 2-8°C to the Microbiology laboratory at the Universitas Gadjah Mada, Special Region of Yogyakarta, Indonesia for further sample processing, i.e., sample preparation, RNA extraction, RT-qPCR, and analysis.

1. Sample Preparation

The wastewater samples, passive samplers, and soil samples were stored at 4°C upon arrival until processing. Sample filtration was conducted as described by others [1], where 50 mL of wastewater or 1,000 mL of recreational, or environmental water was filtered through a 47 mm diameter, 0.45µm pore size, cellulose nitrate high flow electronegative membrane (Sartorius, Germany) using the Microsart vacuum manifold (Sartorius, Germany). This filtration process was performed immediately (~2 hours) once the samples were received at the laboratory**.** For every batch of filtered samples, 100 mL of sterile water was included in the filtration process as the negative control and to monitor the filtration performance.

The passive samplers were dismantled and the filter membrane stored in a 2 mL tube. The collection bag containing the soil samples was thoroughly mixed,then 0.25 grams of soil and 2 mL of DNA/RNA Shield solutions (Zymo Research, USA) were added into 2 mL tubes. All the processed samples from grab wastewater samples, passive samplers, and soil were immediately stored at -80°C until RNA extraction.

1. RNA extraction

The RNA was extracted from samples using the QIAGEN RNeasy PowerMicrobiome Kit (QIAGEN, Germany) following the manufacturer’s instructions, with the exception of replacing the supplied beads with PowerBead Tubes-Garnet beads (QIAGEN, Germany). For every batch of samples processed, a negative extraction control, positive control, and internal control (MS2 bacteriophage) as supplied in the PerkinElmer SARS-CoV-2 Nucleic Acid Detection Kit (RUO) (PerkinElmer) were included in the RNA extraction process to monitor the RNA extraction performance. A 100 μL of phenol–chloroform–isoamyl alcohol (pH 6.5 – 8.0) was added to the PowerBead Tube. Each filter membrane or the 200 μL soil samples were placed into PowerBead Tubes-Garnet beads (QIAGEN, Germany) and then 650 μL PM1–β-ME solution was added. The samples were homogenized at the maximum speed for five minutes using the mini bead beater (Biospec, USA). The RNA extraction samples used 650 μL of the upper layer supplemented with 20 μL of the MS2 bacteriophage internal positive control as supplied in the PerkinElmer SARS-CoV-2 Nucleic Acid Detection Kit (RUO) (PerkinElmer). The subsequent process of RNA extraction followed the QIAGEN RNeasy PowerMicrobiome kit manufacturer’s instructions (QIAGEN, Germany). The RNA was directly used for the RT-qPCR or stored at -80°C until RT-qPCR was performed.

1. RT-qPCR and gene copy estimation

The RT-qPCR using the SARS-CoV-2 Real-time RT-PCR Assay (PerkinElmer®, USA) was used to detect SARS-CoV-2 in the samples. The kit is a multiplex assay that detects the nucleocapsid (N) gene and the open reading frame 1ab (ORF-1ab) gene of the SARS-CoV-2 virus. RT-qPCR assays were performed using a 5 μL of RNA template, with a total reaction volume of 30 μL. Thermal cycling was performed according to the manufacturer’s instruction, as follows, 37 °C for 2 min (1 cycle), 50 °C for 5 min (1 cycle), 42 °C for 35 min (1 cycle) followed by 94 °C for 10 min (1 cycle) and then 45 cycles of 94 °C for 10 s, 55 °C for 15 s, and 65 °C for 45 s. The synthetic SARS-CoV-2 RNA Control 1-MT007544.1 (Twist Bioscience, Australia) was used as the standard curve for quantification, following the manufacturer’s instruction. A ‘positive SARS-CoV-2 detection’ was defined from both ORF1ab and N gene signals or a single gene signal with a cycle threshold (Ct) value ≤40. A ‘non-detection’ was defined as the absence of any gene signal in RT-qPCR or the Ct value higher than 40. The RT-qPCR assay was performed as described by the manufacturer's instruction using the LightCycler 96 instrument along with LightCycler 96 software version 1.1.0.1320 and LightCycler® 480 Multiwell Plate 96, white (Roche, Germany). Ct values were used for calculating SARS-CoV-2 genomic copies per liter (gc/L) in the original sample based on calibration curves. For each sample and primer set (N or ORF1ab), RT-qPCR was performed in duplicate. Therefore, four estimates of the viral RNA concentration were obtained for each sample.

The limit of detection (LOD) for the RT-qPCR assay was determined by the analysis of ten replicates for each dilution of the synthetic SARS-CoV-2 RNA Control 1-MT007544.1 (Twist Bioscience, Australia) analysed and was defined as the lowest number of copies of the N gene target and ORF1ab gene that could be detected in 80% of the replicates tested. The LOD was expressed as the lowest detectable concentration of the N gene target and ORF1ab gene in a sample based on the equivalent volume of sample analysed in each RT-qPCR assay, not adjusting for any potential loss through the processing of the sample or any potential inhibition of the RT-qPCR assay [2]. The final LOD calculation was 5 gene copies per reaction.

For qualitative results, positive samples were defined when at least one of the duplicates was positive. For gene copy estimation in quantitative results, geometric means were calculated from both duplicates. If both duplicates were positive, the geometric mean of both duplicates was reported. If only one duplicate was positive, the LOD value was used to replace the negative value, and then the geometric mean from both duplicates was calculated.

In order to report the actual value of SARS-CoV-2 RNA, we calculated the recovery efficiency. In each RT-qPCR run, multiple SARS-CoV-2 RNA controls, an MS2 phage control (to determine the RNA recovery efficiency and as internal control) of different known concentrations, and negative control were included. The calculation for recovery efficiency is copies recovered/copies spiked × 100 [3].

The reagent volumes used for the RT-qPCR are outlined in S2 Table. The thermocycler parameters are presented in S3 Table. The standard curves generated for both the N and ORF1ab gene targets are presented in S4 Fig. For additional details regarding the RT-qPCR kit used, please refer to the manufacturer’s guide.

**S2 Table. RT-qPCR mastermix used in this study.**

| **Reagent** | **Volume per reaction** |
| --- | --- |
| nCoV Reagent A | 7.5 µL |
| nCoV Reagent B | 1.5 µL |
| nCoV Enzyme Mix | 1 µL |
| UltraPure DNase/RNase free water | 15 µL |
| RNA template | 5 µL |

**S3 Table. Complete thermocycling parameters.**

| **Step** | **Temperature** | **Time** | **Number of Cycles** |
| --- | --- | --- | --- |
| 1 | 37^o^C | 2 minutes | 1 |
| 2 | 50^o^C | 5 minutes | 1 |
| 3 | 42^o^C | 35 minutes | 1 |
| 4 | 94^o^C | 10 minutes | 1 |
| 5 | 94^o^C | 10 seconds | 45 |
|  | 55^o^C | 15 seconds |  |
|  | 65^o^C* | 45 seconds |  |

*Collect fluorescent signal during the final 65^o^C


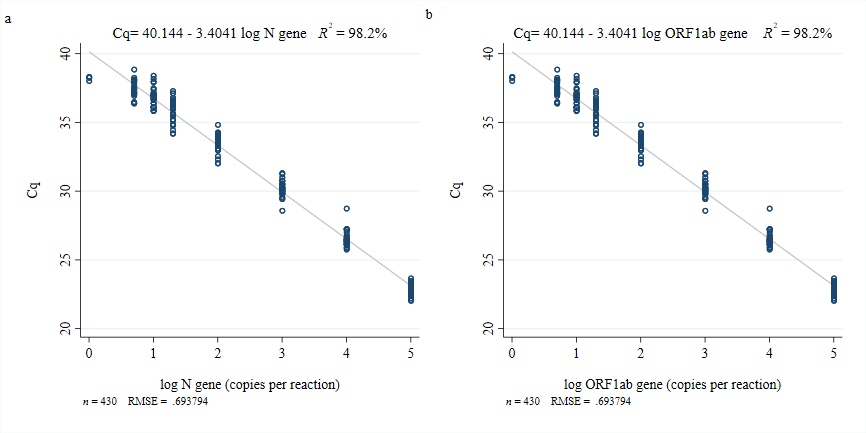


**S4 Fig. Standard curve (excluding values that were below detection).** S4a. N gene. S4b. ORF1ab gene. Dots represent individual standard curve values from each batch of RT-qPCR. Black line represents the slope of all standard curve values.

**References**

1. Ahmed W, Angel N, Edson J, Bibby K, Bivins A, O’Brien JW, et al. First confirmed detection of SARS-CoV-2 in untreated wastewater in Australia: A proof of concept for the wastewater surveillance of COVID-19 in the community. Science of The Total Environment. 2020 Aug 1;728:138764.

2. Black J, Aung P, Nolan M, Roney E, Poon R, Hennessy D, et al. Epidemiological evaluation of sewage surveillance as a tool to detect the presence of COVID-19 cases in a low case load setting. Sci Total Environ. 2021 Sep 10;786:147469.

3. Habtewold J, McCarthy D, McBean E, Law I, Goodridge L, Habash M, et al. Passive sampling, a practical method for wastewater-based surveillance of SARS-CoV-2. Environmental Research. 2022 Mar 1;204:112058.
