# Supplementary material for: Wastewater-based epidemiology surveillance as an early warning system for SARS-CoV-2 in Indonesia
Source: PLoS One. 2024 Jul 18;19(7):e0307364. doi: 10.1371/journal.pone.0307364 (PMC11257287; doi:10.1371/journal.pone.0307364)
Supplement: S1 Table — (DOCX) [file pone.0307364.s005.docx]

**S1 Table. Summary of sample sources**

| **Sample source** | **Number of samples collected** | | |
| --- | --- | --- | --- |
|  | **Delta wave**  **(<7 January 2022)** | **Omicron wave**  **(>18 January 2022)** | **All periods** |
| Manhole (grab) | 621 (50.6%) | 140 (39.4%) | 761 (48.1%) |
| Central WWTP (passive) | 24 (1.96%) | 19 (5.4%) | 43 (2.7%) |
| Community WWTP (passive) | 105 (8.6%) | 40 (11.3%) | 145 (9.2%) |
| Soil (grab) | 119 (9.7%) | 0 (0%) | 119 (7.5%) |
| NST (passive) | 262 (21.4%) | 136 (38.3%) | 398 (25.2%) |
| River (grab) | 96 (7.8%) | 20 (5.6%) | 116 (7.3%) |
| Total | 1,227 | 355 | 1,582 |

WWTP, wastewater treatment plant; NST, near-source tracking.
